# Editorial Notes

**Published:** 1888-12

**Authors:** 


### HOMŒOPATHIC PHYSICIAN

A MONTHLY JOURNAL OF

HOMŒOPATHIC MATERIA MEDICA AND CLINICAL MEDICINE.

"If our school ever give up the strict inductive method of Hahnemann, we are lost, and deserve only to be mentioned as a caricature in the history of medicine."—CONSTANTINE HERING.

Vol. VIII. DECEMBER, 1888. No. 12.

### EDITORIAL NOTES.

THE PAST YEAR: A review of the medical history of the year now drawing to its end reveals a gratifying increase in the interest taken in the study of the materia medica. Many clubs have been formed, in different places, for the study of the *Organon* and the homœopathic materia medica; these clubs have so far given evidence of great future usefulness, and it is to be hoped the members will not allow their zeal to flag.

A few years ago there was scarcely one homœopathic college in this broad land which made even a pretense at teaching Hahnemann's *Organon*, but now, since this journal began its crusade against these derelict colleges, all of them are trying to teach this indispensable branch of homœopathic medicine. As there are very few homœopathic physicians who really understand the *Organon*, there are of course very few who are qualified to properly teach it; still, even an attempt at teaching it is better than entire neglect. In former years physicians have graduated at these colleges who declared they had never heard the *Organon* even mentioned; therefore any change from past neglect can scarcely fail to be something of an improvement.

The establishment of a post-graduate course for the study of the *Organon* and the homœopathic materia medica in this city, by Professor Kent, is another step in the right direction, and one of which it is to be hoped many students will avail themselves. The so-called "Hahnemann College," of this city, has classes which are certainly fed on very light diet in these branches; the students of this institution have now an opportunity to learn something of Homœopathy.

We desire to extend our congratulations to our venerable colleague and former teacher, Professor Lilienthal, of San Francisco, who has recently completed his fiftieth year in medicine. Few there are who attain to so many years of medical work, and fewer still are they who use their years to such good purpose as has our venerable friend. Dr. Lilienthal has been, and not inaptly, called the American Jahr, in recognition of his great literary diligence; as editor, author, and teacher Dr. Lilienthal has always shown great ability and unceasing activity. May many years yet be vouchsafed to him, and may his mantle fall upon a worthy successor.

During the past year it has been the misfortune of this journal to lose two of its best friends and ablest supporters, in the death of Drs. Lippe and Fellger. Both were men of rare ability, and physicians of great success; we can only deplore their loss and pray that successors may soon arise to fully occupy their vacant places.

---

THE COMING YEAR: In order to make our coming year's work one of greater usefulness than any of its predecessors, we beg leave to remind our subscribers that each one of them should be a contributor to our pages. The editors do not and cannot make a successful journal, that lies entirely with its contributors. THE HOMŒOPATHIC PHYSICIAN has had an exceptionally able and active corps of contributors, for whose past assistance we return our sincere thanks. We trust the coming year will find *every subscriber a contributor*. Let each physician add his mite to the sum of general knowledge; then true Homœopathy will indeed go forward with a bound.

It has been our rule in the past to limit the size of each monthly issue solely according to the amount of useful, practical papers furnished us. If we have only a limited supply, we publish a smaller number; if we have a larger supply, we issue a large monthly number. We do not publish trash merely to add to our pages.

THE HOMŒOPATHIC PHYSICIAN was started to support true Homœopathy; it is the only journal which has this object for its sole purpose. It is not published to make money; all the money received is spent upon the journal. The more subscribers we have the larger journal we can publish, and greater will be our field of usefulness; each subscriber is therefore interested in securing us at least *one* new subscriber. The trouble taken in canvassing will be paid for in the better and larger journal received. Let each of our present subscribers resolve to write *one paper* for our pages, and also to secure *one new* subscriber for 1889; before the end of the year he will feel himself amply repaid for his labor.

As we have been able in the past few years, not only to pay our current expenses, but also to save a small amount, we give our subscribers the advantage of this success by offering the journal for 1889 for two dollars ($2) to all who send their subscriptions before the 1st of April. Those who delay payment until a later date must pay the old subscription fee of two dollars and fifty cents ($2.50). In making this offer we benefit both our subscribers and ourselves; for it is a great help to us to have our funds in hand early in the year, so we may know how much we will have to rely upon for the year's work. Send us, therefore, at once your two-dollar bill, and tell us when to expect your *new* subscriber and your MSS. E. J. L.

